# Molecular Epidemiology of Human Adenovirus, Astrovirus, and Sapovirus Among Outpatient Children With Acute Diarrhea in Chongqing, China, 2017–2019

**DOI:** 10.3389/fped.2022.826600

**Published:** 2022-03-03

**Authors:** Xiang Tang, Yue Hu, Xiaoni Zhong, Hongmei Xu

**Affiliations:** ^1^Children’s Hosptital of Chongqing Medical University, Chongqing, China; ^2^Ministry of Education Key Laboratory of Child Development and Disorders, National Clinical Research Center for Child Health and Disorders, China International Science and Technology Cooperation Base of Child Development and Critical Disorders, Chongqing Key Laboratory of Child Infection and Immunity, Chongqing, China; ^3^School of Public Health and Management, Chongqing Medical University, Chongqing, China

**Keywords:** human adenovirus, astrovirus, sapovirus, acute diarrhea, genotype

## Abstract

**Objective:**

To investigate the epidemiology of human adenovirus (HAdV), human astrovirus (HAstV), and sapovirus (SaV), children with acute diarrhea in Chongqing, China from 2017 to 2019 were enrolled. Improved surveillance could provide better guidance for diarrhea prevention.

**Methods:**

Between 2017 and 2019, fecal specimens were collected from children <14 years of age presenting with acute diarrhea for treatment at the outpatient department of the Children’s Hospital, Chongqing Medical University. Human HAdV in the fecal specimens was detected by PCR, while RT-PCR was adopted for the detection of HAstV and SaV.

**Results:**

A total of 1,352 fecal specimens were screened in this study. The detection rate of HAdV was 4.44% (60/1352), HAstV was 2.81% (38/1352), and SaV was 1.04% (14/1352). The prevalence of enteric viruses in males was not significantly different to females (*p* > 0.05). We found 96.67% (58/60) of the HAdV-positive cases, 92.11% (35/38) of the HAstV-positive cases, and 100% (14/14) of the SaV-positive cases among the children under 4 years old. HAdV cases were identified throughout the year, while the infection of HAstV peaked from March to May every year. By contrast, SaV was detected in May, July, and from September to December. In total, 41 strains of HAdV-F were identified, including F41 (39/60) and F40 (2/60). Furthermore, A31, B3, B7, C1, C2, C5, and C6 were also detected in the study. In addition, we detected two genotypes of HAstV, HAstV-1 (34/38) and HAstV-5 (4/38), and two genotypes of SaV, GI0.1 (13/14), GI0.2 (1/14).

**Conclusion:**

The enteric viruses HAdV, HAstV, and SaV contribute to the overall burden of diarrhea in Chongqing, especially in children <4 years of age. Two genotypes were identified for HAstV (HAstV-1 and HAstV-5) and SaV (GI.1 and GI.2) with an additional nine genotypes detected in HAdV cases. While the F41 HAdV strain was predominant, HAdV-A31 was also detected in 10% of cases. The study results along with continuous surveillance of enteric viruses will aid in the design and implementation of future enteric vaccines and diarrhea mitigation strategies.

## Introduction

Since 1990, mortality rate of children with diarrhea has been significantly decreased because of the improvements in water, sanitation, and hygiene (1, 2). However, diarrhea is still the world’s second leading infectious cause of death in children under 5 years old. According to a previous report, approximately 530,000 children under the age of 5 died from diarrheal diseases in 2017 (1). Therefore, monitoring diarrheal diseases is still a priority. Watery diarrhea and vomiting can cause dehydration. Delays of admission to a hospital, incorrect or imprompt treatment may increase disease severity, which may subsequently result in death. A diarrheal episode could cause serious damages to the bodies of young children, which would take a long time to restore normal nutrition. Thus, the growth and development of children would be affected (3). Acute diarrhea could be caused by many factors, among which viral infection is the most common one (4). Previous studies have identified that rotavirus (RV), norovirus (NoV), human adenovirus (HAdV), sapovirus (SaV), and human astrovirus (HAstV), are major viral etiologies of diarrheal illness (2). RV remains the leading cause of severe gastroenteritis worldwide. However, the introduction of RV vaccine could provide effective protection for children (5). Therefore, other diarrheal viruses, such as HAdV, HAstV, and SaV have attracted more attention (6). Currently, routine diagnostics are performed for RV and NoV using enzyme immunoassays (EIAs), but surveillance for HAdV, HAstV, and SaV in China is still limited. Therefore, additional molecular screening for these viruses was implemented at a children’s hospital outpatient department for three years in this study, which would be benefit in understanding of epidemiology in SaV, HAstV, and HAdV.

Human adenovirus is a non-enveloped linear double-stranded DNA virus detected in various glands, such as the tonsils and mesenteric lymph nodes. HAdV has been divided into over 100 serotypes (HAdV Working Group, updated April, 2021^1^), which can be further grouped into seven genotypes (A–G). Different genotypes may cause different symptoms. In addition to gastroenteritis, HAdV also causes acute respiratory illness, conjunctiva, hemorrhagic cystitis, hepatitis, hemorrhagic colitis, pancreatitis, nephritis, and meningoencephalitis (7). It has been proposed that both HAdV41 and HAdV40 belong to the subgroup of F, which are known as enteric adenoviruses (EAds) (8, 9).

HAstV was first identified in 1975 by Appleton and Higgins during electron microscopic examination of fecal specimens, obtained from children with acute gastroenteritis. HAstV are non-enveloped single-stranded RNA (ssRNA) viruses with genome size of 6.2–7.7 kb, which contains three open reading frames (ORFs, that are, ORF1a, ORF1b, and ORF2). ORF1a and ORF1b encode some non-structural proteins, such as RNA-dependent RNA polymerase whilst ORF2 encodes the capsid protein precursor (10). Currently, HAstV strains are categorized into eight classic genotypes (HAstV-1–HAstV-8), and two novel genotypes (HAstV-MLB and HAstV-VA/HMO). The majority of childhood diarrhea is caused by the classic HAstV genotypes and there is still uncertainty about the role of HAstV-MLB and HAstV-VA/HMO in diarrheal disease (11).

Sapovirus, a genus in the Caliciviridae family alongside NoV, is increasingly recognized as an important cause of childhood diarrhea. Overall, 19 genogroups (GI–GXIX) have been described in SaV, with genogroups GI, GII, GIV, and GV limited to humans (including GI.1–7; GII.1–8; GIV.1; and GV.1–2) (12). SaV was rarely studied compared to NoV in the past. Recently, it has been shown that SaV infection is widely distributed. SaV is responsible for both sporadic cases and outbreaks of acute diarrhea.

As a city located on the intersection of two rivers (Yangtze River and Jialing River), Chongqing has extreme seasonal variations displayed in temperature (“hot summer and cold winter”) compared to other cities. In the present study, three diarrheal viruses in pediatric patients in Chongqing were monitored throughout the year for the early detection of new variants in the population. Therefore, the improved surveillance could provide better guidance for diarrhea prevention.

## Subjects and Methods

### Subjects

In total, 1,352 fecal specimens were collected by healthcare professionals from January 1, 2017 to December 31, 2019 at the Clinical Laboratory Center of The Affiliated Children’s Hospital of Chongqing Medical University. All the collected samples were assessed in the present study. The inclusion criteria for collected samples were as follows: frequent bowel movements (three or more per day); loose, explosive, and watery stools; WBC (White blood cells) <10 HP-1 (high power field)examined by microscopic; exclusion of both mucous stools and bloody purulent stools; and less than 2 weeks of disease duration (since onset). The collected samples were stored at -20°C immediately, and repeated freezing and thawing was avoided. Written consent was obtained from the parents of all pediatric children. General information and the disease conditions of the patients were recorded. This study was approved by the medical ethics committee of the Children’s Hospital of Chongqing Medical University [File No.2020 ethical review (research) No.154].

### Methods

#### Main Reagents

QIAamp Viral RNA Mini Kits were purchased from QIAGEN GmbH (Hilden, Germany). SuperScript III First-Strand Synthesis System reverse transcriptase was purchased from Invitrogen Inc. (Carlsbad, CA, United States). ExTaqDNA polymerase was purchased from Takara Biotechnology (Dalian) Co., Ltd. (China).

#### Detection of HAdV, HAstV, and SaV

Viral RNA was extracted using the QIAamp^®^ Viral RNA Mini Kit, while reverse transcription was performed using the SuperScript^®^ III First-Strand Synthesis System for RT-PCR. All procedures were conducted in accordance with the instructions provided in the kits. In the present study, all samples were tested for AdV, SaV, and AstV using reverse transcription-polymerase chain reaction (RT-PCR), or PCR. Gene region of hexon was amplified for detection of HAdV, while gene region of capsid (ORF2) was amplified for detection of HAstV. Capsid protein VP1 was amplified for detection of SaV. The primer pairs selected to detect HAdV, HAstV and SaV were named as Ad1 and Ad2, Mon269 and Mon270, SLV5317 and SLV5749, respectively. The size of the amplicons were 482 bp for HAdV, 449 bp for HAstV, and 434 bp for SaV. These primer sequences were designed as previously reported (13, 14).

The PCR reaction conditions were as follows: pre-denaturation at 94°C for 5 min, followed by 35 cycles of 94°C denaturation for 30 s, 55°C annealing for 30 s, and 72°C extension for 1 min, followed by a final extension at 72°C for 7 min, and termination at 4°C. Agarose gel electrophoresis (1.5%) was performed for analysis of the PCR products.

#### Sequence Analysis

All virus-positive (HAdV, HAstV, and SaV) samples were subjected to gel recovery and purification before sent to Invitrogen Biotechnology (Shanghai) Co., Ltd., for sequencing. The sequencing results were processed by DNAstar software. The obtained sequences were aligned with sequences retrieved from the GenBank database using BLAST searches. A phylogenetic tree was established by MEGA 7.0 software with Kimura’s 2-parameter to calculate the genetic distance, while the neighbor-joining method with boot-strap was deployed.

GeneBank database was used to obtain all reference sequences. The gene sequences described in the present study have been deposited in the GenBank database under the accession number of OL681900-OL682011.

#### Statistical Analysis

Statistical significance of the obtained data was determined by Excel and SPSS 23.0 software. Measurement data was denoted in the form of (x ± s), while counting data was subjected to the chi-square (χ^2^) test. Differences at level of *P* < 0.05 was considered as statistically significant.

## Results

In this study, a total of 1,352 stool samples collected from children with acute diarrhea were enrolled from January 1, 2017 to December 31, 2019 (including 444 samples in 2017, 475 samples in 2018, and 433 samples in 2019). Amongst all the samples, stool was sampled from 802 boys and 550 girls. All of the enrolled children had been diagnosed with acute diarrhea at the Children’s Hospital of Chongqing Medical University, who were followed with attendance as outpatients. The ages of the enrolled patients ranged from 10 days to 166 months, with an average of 16.34 ± 17.83 months.

Among these 1,352 fecal samples, 8.28% (112/1352) were infected with the three enteric viruses. A total of 60 cases [4.44% (60/1352)] with HAdV were detected in the 3 years. Specifically, the HAdV-positive rate was 4.95% (22/444) in 2017, 5.05% (24/475) in 2018, and 3.23% (14/433) in 2019. We also identified 38 cases with HAstV (2.81%, 38/1352), with a detection rate of 3.60% (16/444) in 2017, 2.74% (13/475) in 2018, and 2.08% in 2019 (9/433). In addition, 14 cases (1.04%, 14/1352) were positive for SaV, with a SaV detection rate of 1.80% (8/444) in 2017, 0.63% (3/475) in 2018, and 0.69% (3/433) in 2019. The detection rates of the three viruses fluctuated in each year, however, the differences were not statistically significant (χ^2^ HAdV = 2.185, *P* = 0.335; χ^2^ HAstV = 1.881, *P* = 0.390; χ^2^ SaV = 3.301, *P* = 0.165).

Most cases occurred in children <4 years with 96.67% (58/60) of HAdV infection, 92.11% (35/38) of HAstV infection, and 100% (14/14) of SaV infection detected. Among all age groups, children 25–36 months showed the highest HAdV-detection rate [9.30% (8/86)]. In contrast, children 49–60 months and >60 months showed the highest HAstV infection rate (4.54%). However, only one case infected with HAstV were detected in the children 49–60 months and two cases infected with HAstV were detected in the children in the >60 months category. Children 13–18 months had the highest rate (2.34%, 6/256) of SaV detected. Taken together, the total positive rate of the three viruses gradually increased from six months to three years old, and gradually decreased after three years old. In contrast, the positive rate was relatively low in children under six months of age (Table 1).

**TABLE 1:** Distribution of viral pathogens in 1352 children with acute gastroenteritis different age group in Chongqing.

Age group (months)	Samples	Total *n* (%)	HAdV *n* (%)	HAstV *n* (%)	SaV *n* (%)	Mixed infection *n* (%)
0–6	312	9 (2.88)	3 (0.96)	6 (1.92)	0 (0)	0 (0)
7–12	444	35 (7.88)	21 (4.73)	12 (2.7)	2 (0.45)	1(0.23)^a^
13–18	256	26 (10.16)	13 (5.08)	7 (2.73)	6 (2.34)	0 (0)
19–24	131	17 (12.98)	9 (6.87)	5 (3.82)	3 (2.29)	2(1.53)^b^
25–36	86	13 (15.12)	8 (9.3)	3 (3.49)	2 (2.33)	0 (0)
37–48	57	7 (12.28)	4 (7.02)	2 (3.51)	1 (1.75)	0 (0)
49–60	22	1 (4.55)	0 (0)	1 (4.55)	0 (0)	0 (0)
>60	44	4 (9.09)	2 (4.55)	2 (4.55)	0 (0)	0 (0)
Total	1352	112 (8.28)	60 (4.44)	38 (2.81)	14 (1.04)	3 (0.22)

*n(%) denotes number and consituent ratio. ^a^Tri-viral mixed infection of HAdV-F41 + HAstV-1 + GI0.2. ^b^Bi-viral mixed infection occurred in two cases.*

**FIGURE 1:**
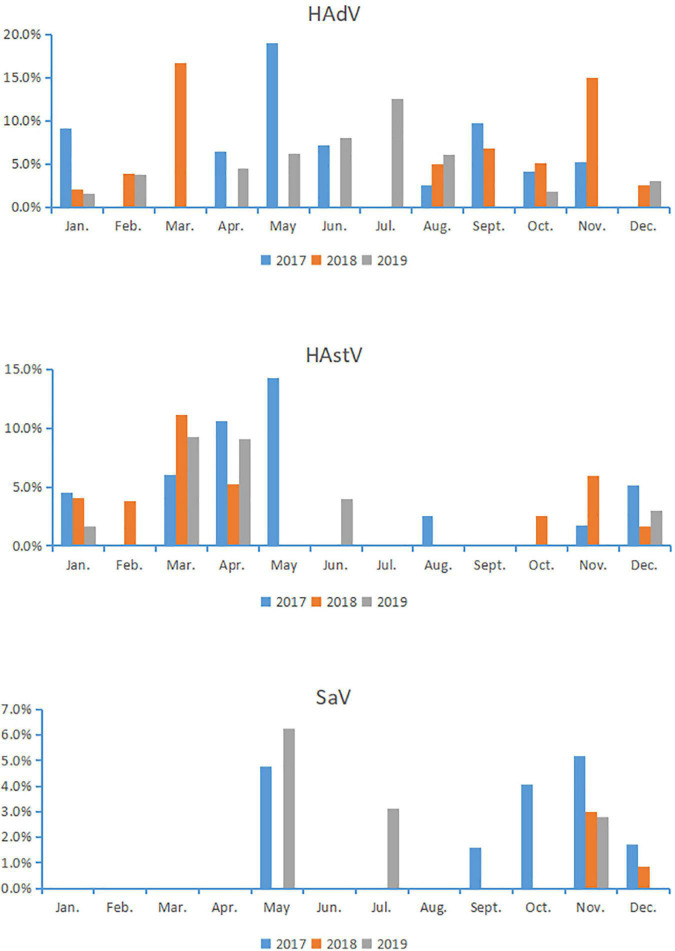
Monthly distribution of sapovirus, astrovirus, and human adenovirus detection.

Human adenovirus infections were identified throughout the year, accompanied with a peak infection in May (19.05%) of 2017, two peaks in March (16.67%) and November (14.93%) of 2018, and one peak in July (12.50%) of 2019. These results indicate that there was no fixed season for HAdV infection in Chongqing. HAstV were detected in all months except July and September, with a peak infection in May (14.3%) of 2017, a peak in March (11.1%) of 2018, a peak in March (9.30%) of 2019, suggesting that HAstV was prevalent in spring in Chongqing. SaV was detected in May, July, September to December, with no detection in the remaining months. Notably, infections of SaV were mainly around May and November, suggesting that SaV was prevalent at the turn of autumn and winter or the turn of spring and summer in Chongqing (Figure 1).

The most common clinical symptom of the patients infected with HAdV was diarrhea (100.0%, 60/60), followed by vomiting (50%%, 30/60), fever (36.67%, 22/60), and cough (45%, 27/60). The common clinical symptoms of HAstV-infected patients were diarrhea (100.0%, 38/38), fever (28.95%, 11/38), vomiting (50%, 19/38) and cough (50%, 19/38). In addition, the common clinical symptoms of classic SaV-infected patients were diarrhea (100.0%, 14/14), fever (7.14%, 1/14), vomiting (64.29%, 9/14), and cough (28.57%, 4/14). The occurrence of diarrhea and vomiting was significantly higher in SaV-positive cases (64.29%; 9/14) compared to SaV-negative cases (47.38%; 634/1338; *P* = 0.002) (Table 2).

**TABLE 2:** Clinical symptoms of diarrhea children infected with and without HAdV, HAstV and SaV.

Symptoms	HAdV n (%)		HAstV n (%)		SaV n (%)	
	Positive	Negative	Positive	Negative	Positive	Negative
diarrhea	60 (100)	1292 (100)	38 (100)	1314 (100)	14 (100)	1338 (100)
fever	22 (36.67)	423 (32.74)	11 (28.95)	434 (33.03)	1 (7.14)	444 (33.18)
vomiting	30 (50.00)	613 (47.45)	19 (50.00)	624 (47.49)	9 (64.29)	634 (47.38)^a^
cough	27 (45.00)	512 (39.63)	19 (50.00)	520 (39.57)	4 (28.57)	535 (39.99)

*^a^The difference between infected and uninfected SaV group was statistically significant when the clinical symptoms of vomiting (P = 0.002).*

**FIGURE 2:**
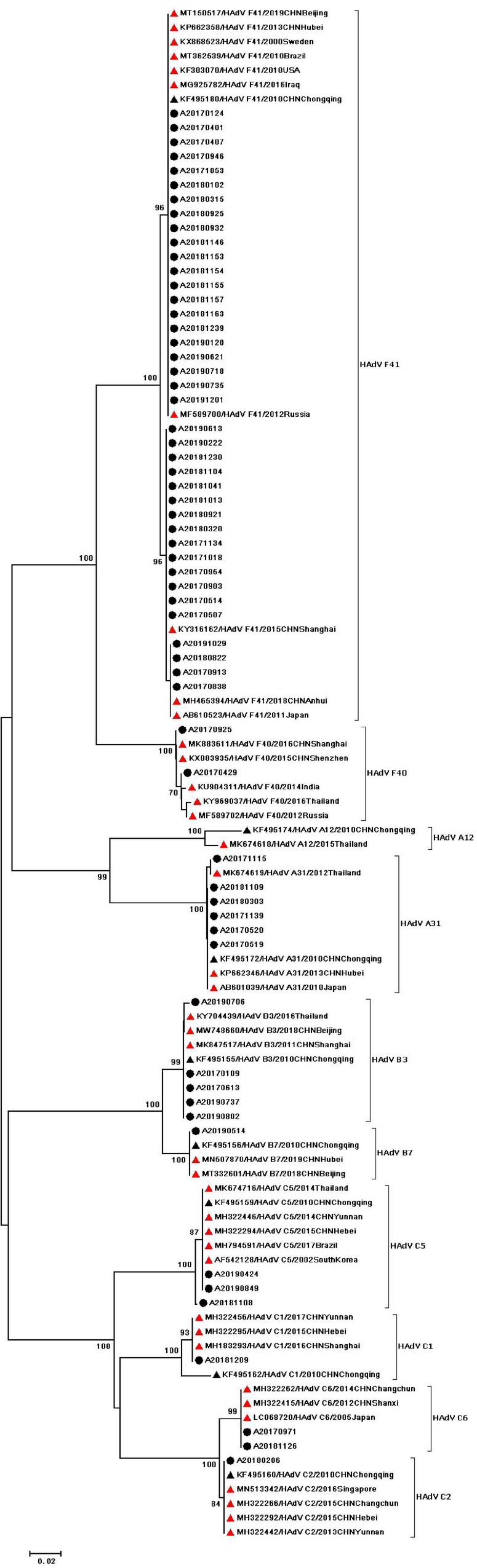
Phylogenetic tree of HAdV detected in Chongqing based on partial nucleotide sequences (482 bp) of the exon gene. The dendrogram was constructed by the maximum likelihood method with the Kimura two-parameter model in MEGA 7.0. Numbers at each node represents bootstrap values obtained with 1,000 replicates (>70 are shown). The scale bar represents the unit for the expected number of substitutions per site. Strains from the present study (designed A/year/month/number) are labeled with a black circle. Red triangles indicate reference strains (including accession number, genotype, year, and location). Black triangles indicate previously reported strains from Chongqing (including accession number, genotype, year, and location).

**FIGURE 3:**
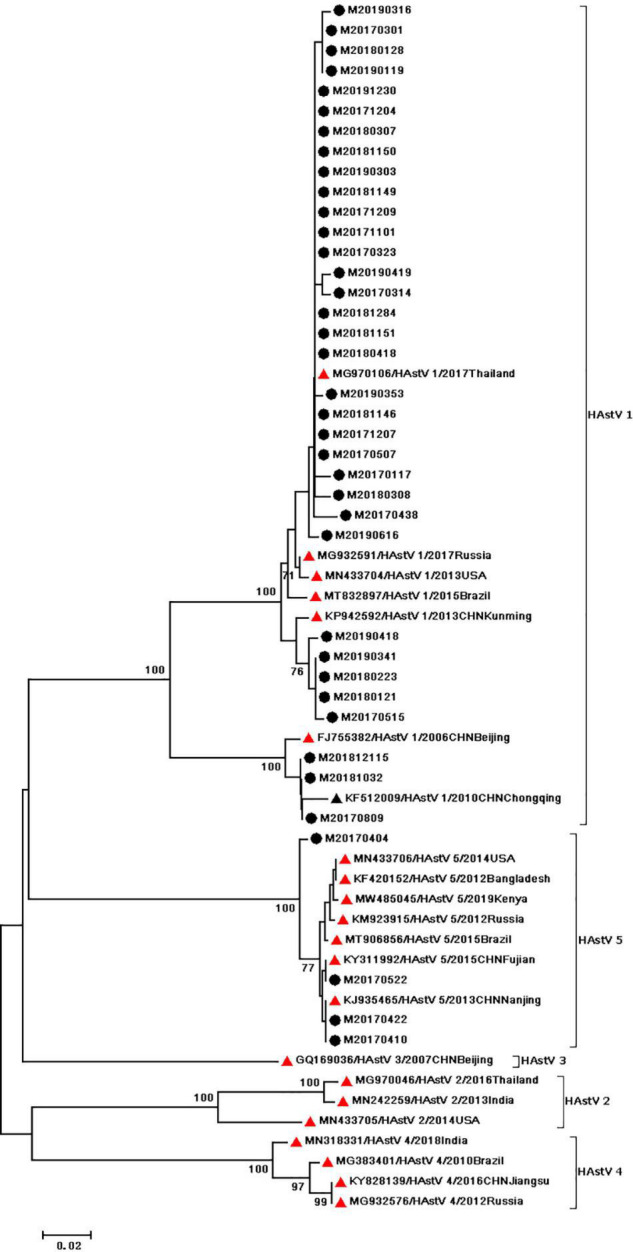
Phylogenetic tree of HAstV detected in Chongqing based on the 449bp-partial capsid (ORF2) sequence. The dendrogram was constructed by the maximum likelihood method with the Kimura two–parameter model in MEGA 7.0. Numbers at each node represents bootstrap values obtained with 1,000 replicates (>70 are shown). The scale bar represents the unit for the expected number of substitutions per site. Strains from the present (designed M/year/month/number) and previous(including accession number, genotype, year, and location) study are labeled with a black circle and black triangles. Red triangles indicate reference strains (including accession number, genotype, year, and location).

**FIGURE 4:**
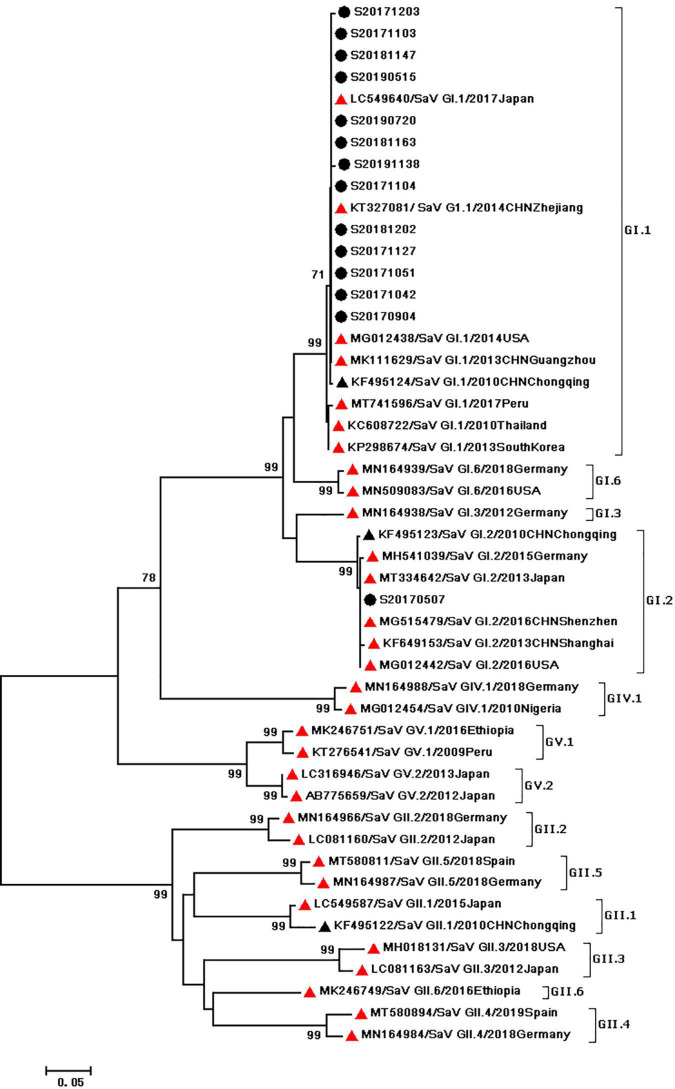
Phylogenetic tree of SaV detected in Chongqing based on the 434bp–partial capsid sequence. The dendrogram was constructed by the maximum likelihood method with the Kimura two–parameter model in MEGA 7.0. Numbers at each node represents bootstrap values obtained with 1,000 replicates (>70 are shown). The scale bar represents the unit for the expected number of substitutions per site. Strains from the present (designed S/year/month/number) and previous(including accession number, genotype, year, and location) study are labeled with a black circle and black triangles. Red triangles indicate reference strains (including accession number, genotype, year, and location).

During the study period, four subgroups of HAdV were detected, including HAdV-A (6/60,10%), HAdV-B (6/60,10%), HAdV-C (7/60,11.67%), and HAdV-F (41/60,68.33%). HAdV-F41 was the most common genotype [65.00% (39/60)] among HAdV cases, followed by A31 (6/60,10%), B3 (5/60,8.3%), C5 (3/60,5.0%), F40 (2/60,3.3%), C6 (2/60,3.3%), B7 (1/60,1.67%), C1 (1/60,1.67%), and C2 (1/60,1.67%). As two causative factors of respiratory infection, HAdV-B and HAdV-C were associated with the respiratory tract symptoms in 7 out of 13 pediatric patients who were infected with HAdV-B or HAdV-C. Phylogenetic analysis showed two clusters of F41 strains circulating in this study. Two F40 strains were detected in 2017, and no A31 strain were detected in 2019 (Figure 2). Two different genotypes of HAstV (HAstV-1 and HAstV-5) were identified among 38 samples, while HAstV-1 (34/38,89.47%) was the predominant genotype. Phylogenetic analysis revealed three clusters of HAstV-1 strains. The major cluster (26 strains) was found to be closely related to the reference strain previously isolated from Thailand (HQ398856) in 2017, which showed 98.57–99.72% nucleotide sequence identity. Additionally, four HAstV-5 strains were detected in 2017 (Figure 3). Two genotypes of SaV were detected, including GI0.1 (13/14,92.9%) and GI0.2 (1/14,7.1%), with one GI0.2 strain was detected in 2017. In addition, the cluster with 13 strains was found to be closely related to the reference strain isolated from chongqing (KF495124) in 2010 (that is 99.04–99.36% nucleotide sequence identity) (Figure 4).

Among the 1352 specimens, there were three cases of mixed infection, which were caused by HAdV and other viruses concomitantly. Bi-viral mixed infections were also detected, which occurred in two cases, including HAdV-F41 + HAstV-1 and HAdV-F41 + GI0.1. We also detected the Tri-viral mixed infection, which occurred in one case (HAdV-F41 + HAstV-1 + GI0.2). All the three cases displayed watery stool. However, one case showed none other clinical symptoms, one showed low-grade fever and the other showed respiratory tract infection. The three cases of mixed infection ranged in age from 12 months to 21 months, which suggests that the mixed infection was prevalent under 2 years old.

## Discussion

Acute infectious diarrhea in children is mainly caused by viruses, while RV and NoV are the two most common viruses (15). HAdV, HAstV, and SaV contributed to the overall diarrheal burden in China. This study mainly monitored the infection of the above three viruses in children exhibiting acute diarrhea from 2017 to 2019 in Chongqing, and all the basic data would provide a theoretical foundation for formulating a strategy to prevent and control viral diarrhea.

In Chongqing, 4.44% of pediatric patients with acute diarrhea were diagnosed with HAdV infection during 2017 and 2019, which was close to the HAdV prevalence in Hangzhou (3.1%, 2017–2018) (16) and Shanghai (3.47%, 2017–2018) (17) reported in recent studies of pediatric diarrhea with HAdV infection in China. HAdV infections are more prevalent in pediatric patients with diarrhea in African countries such as Nigeria (19.3%) (18) and Gabon (19.6%) (19), suggesting a regional difference in HAdV prevalence and correlations of HAdV prevalence with economic development and hygiene conditions. In Chongqing, F41 was more frequent than F40, which was consistent with the findings reported by other studies in Chongqing and other regions in China (15–17, 20, 21). Conversely, HAdV-40 was detected more frequently than F41 in Kolkata, India although the study period was different (2013–2014) (9). HAdV-A was also associated with diarrhea (8) and we detected six A31 strains in this study, which was the second predominant genotype followed by F41, which was similar to the results of a study in Beijing (21). This demonstrated that A31 was an important genotype causing acute diarrhea in pediatric patients. Additionally, 13 strains of HAdV-B and HAdV-C were detected in the present study. Meanwhile, Qiu et al. (22) reported that B3 was a high-risk factor for diarrhea through a comparison of the stool detection rate between pediatric patients with diarrhea and healthy children after the exclusion of the possibility of co-infection with other intestinal pathogens. Gelaw et al. (23) and Kumthip et al. (24) reported that pediatric patients with diarrhea had a higher stool detection rate of HAdV-C than that of HAdV-F, while the HAdV subgroup C was the most prevalent genotype in both Northwest Ethiopia and Thai. However, it remains unknown whether diarrhea is directly caused by HAdV-B or HAdV-C. A possible reason for the coincidental detection of HAdV-B or HAdV-C in stool could be the presence of an asymptomatic elsewhere in the body or shedding of the virus after a recent respiratory, ocular or urinary tract infection. Therefore, more work will be required to establish the association in future.

The present study showed that during the year from 2017 to 2019, the overall HAstV-positive rate in Chongqing was 2.81% in pediatric patients with acute diarrhea, similar to that in Thai (2.6%) (25). The HAstV infection rates were slightly higher in Shanghai (5.22%) (17) and eight low-income countries (5.6%) in Asia, Africa, and South America (e.g., Bangladesh, Tanzania, Peru) (26). In Japan, the HAstV infection rate was 16.4% (27), markedly higher than the results of this study. Mon269 and Mon270 were used for nucleotide sequence amplification in the region of ORF2 of HAstV to identify the genotype of HAstV1-8. Consequently, none of novel HAstV (e.g., HAstV-MLB, HAstV-VA/HMO) were detected in this study. In the present study, two genotypes, namely HAstV-1 and HAstV-5, were detected. HAstV-1 was the predominant strain in our study. In contrast, HAstV-2 was detected in Chongqing in 2014 (20). HAstV-1 has been recognized as a predominant circulating genotype worldwide, followed by HAstV-2–5 and HAstV-8, whereas HAstV-6 and HAstV-7 are rarely detected (10). Japanese researchers reported that primers SF0073 and SF0076 could simultaneously detect both classic and novel HAstVs, which could yield a higher positive rate (16.4%). However, it is still controversial whether the novel HAstVs could result in diarrhea (11). In fact, only 4.8% of the samples were classic HAstV strains in Japan. Moreover, Japanese researchers have demonstrated that over 50% of all HAstV-MLB infections occurred in parallel with both RV and NoV, which resulted in the difficulty in the identification of the primary pathogen (27). In this study, infection of HAstV occurred most frequently during March and May, while HAstV-5 infection was not detected until April and May, indicating that HAstV infections might be epidemic in the spring. It had been reported that HAstV infection was prevalent in January, April and November to December in Chongqing in 2014 (20). Therefore, the infection of HAstV might be associated with the low environmental temperature, thus displayed a typical winter/spring seasonality. In addition, our results were also consistent with the results observed in Shanghai in 2015-2016, which also displayed a typical winter/spring seasonality (28). However, the infection peaks of HAstV varied in different years in this study, and the seasonality of HAstV infections in Chongqing needs to be further monitored.

The frequency of SaV is variable across geographic locations and age groups. However, SaV has been detected in 1–17% of diarrhea in various settings (12). Nevertheless, this study indicated that the SaV prevalence was not high in Chongqing, with an infection rate of 1.04%, which was close to the reported rate of 0.69% in eight cities during 2012–2014 in China (29). In Chongqing, SaV infections occurred more frequently in May or November. Likewise, in the Netherlands, SaV was most commonly detected in the winter and spring (30). Infants aged 7–48 months were affected by the virus. In this study, vomiting was a typical clinical symptom of SaV infection, whereas fever was rarely observed (Table 2). Similarly, a birth cohort in Peru reported that 40% children infected with SaV diarrhea experienced vomiting and 10% experienced fever (31). In surveillance studies of children with SaV diarrhea, the GI.1 genotype was common ranging in prevalence from 10 to 63%, followed by GI.2 and GII.1 (12, 32). Moreover, GI genotypes were prevalant during previous epidemics in Chongqing (15, 20). Notably, SaV outbreaks have been reported in high-income countries, particularly in those possessing a large child population (33, 34). Besides, SaV outbreaks occur frequently in China (35, 36). Therefore, SaV outbreak surveillance deserve attention in future study.

In this study, we also detected three mixed infection cases. However, we found no significant difference between the mixed infection cases and the non-mixed infection cases in terms of the severity of the disease. We also noted that the mixed infection occurred among young infants and toddlers. Our results were in consistent with previous reports (37, 38).

## Conclusion

The enteric viruses, HAdV, HAstV, and SaV, contribute to the overall burden of diarrhea in outpatient cases in Chongqing, especially in children <4 years of age. HAdV was found year-round while HAstV and SaV displayed some seasonality. Predominant circulating viral strains included HAdV-F41, HAstV-1, and SaV GI.1 with the study contributing sequence data to global molecular epidemiology of enteric viruses. Continuous surveillance of enteric viruses is recommended to aid in the design and implementation of future diarrhea mitigation strategies and enteric vaccines.

## Limitation of This Study

Only three enteric viruses were investigated during the study and expanded molecular testing for RV, NoV as well as enteric bacteria and parasites would provide a holistic picture of the pathogens contributing to diarrheal disease in Chongqing. In addition, the primers used to detect HAstV strains may bias the results, detecting only classic or recombinant HAstV strains and not novel HAstV.

## Data Availability Statement

Publicly available datasets were analyzed in this study. This data can be found here: OL681900-OL682011.

## Ethics Statement

The studies involving human participants were reviewed and approved by Institutional Review Board of Children’s Hospital of Chongqing Medical University. Written informed consent to participate in this study was provided by the participants’ legal guardian/next of kin. Written informed consent was obtained from the minor(s)’ legal guardian/next of kin for the publication of any potentially identifiable images or data included in this article.

## Author Contributions

XT and YH performed the experiments, analyzed the data, and wrote the manuscript. H-MX designed the study and revised the manuscript. X-NZ coordinated the study and analyzed the data. All authors approved the final version of the manuscript.

## Conflict of Interest

The authors declare that the research was conducted in the absence of any commercial or financial relationships that could be construed as a potential conflict of interest.

## Publisher’s Note

All claims expressed in this article are solely those of the authors and do not necessarily represent those of their affiliated organizations, or those of the publisher, the editors and the reviewers. Any product that may be evaluated in this article, or claim that may be made by its manufacturer, is not guaranteed or endorsed by the publisher.

## Footnotes

1
http://hadvwg.gmu.edu
